# A Usability Study of MoCA Solo, a Digital AI‐Assisted Version of the MoCA Test

**DOI:** 10.1002/alz70857_097196

**Published:** 2025-12-24

**Authors:** Johanna Gruber, Joana Krieger, Richard Jansen, Willem Huijbers, Kacylia Huijbers Pistoia, Murray Gillies, Ziad Nasreddine

**Affiliations:** ^1^ MoCA Clinic and Institute, Greenfield Park, QC, Canada; ^2^ MoCA Cognition, Greenfield Park, QC, Canada

## Abstract

**Background:**

MoCA Solo from Montreal Cognitive Assessment (MoCA) is a digital version of the MoCA test. It is self‐administered, with AI Assistance, by the patient via an application on a tablet device with minimal supervision. The MoCA test has become the gold standard for detecting cognitive impairment in both clinical and research settings worldwide. However, the traditional paper‐and‐pencil test presents challenges in administration and scoring due to its sensitivity to human variation and error. The MoCA Solo, with automated administration, data collection and AI assisted scoring, would address these limitations and could enhance accessibility, efficiency and reliability. This study aims to assess whether older adults with a range of cognitive impairment levels can complete the MoCA Solo with minimal supervision.

**Method:**

40 older adults will be recruited at the MoCA Clinic as well as a senior residence in Montreal, Quebec. Participants’ cognitive level will range between mild dementia to normal cognition (MoCA Score 11‐30). Eligibility criteria will include: fluency in English or French, adults aged 50 years or over, at least 6 years of formal education, MoCA score ≥11. Participants will complete the MoCA Solo independently followed by a user experience questionnaire. The test completion rate will be evaluated at each level of cognitive impairment. User experience feedback will also be reviewed for future improvements in the application.

**Result:**

Results from the 40 participants will be available by July 2024.

**Conclusion:**

The MoCA Solo is emerging as a reliable and efficient tool for assessing cognitive impairment in older adults. Preliminary findings will inform its accessibility and usability, offering insights into its feasibility for widespread use in clinical and research settings across varying levels of cognitive impairment.

